# Obituary Notices

**Published:** 1864-02

**Authors:** 


### OBITUARY NOTICES.

Dr. C. H. Cleveland, of Cincinnati, a former contributor to this journal, died in the city of Memphis, on the 1st of December last, of Congestive Pneumonia, aged 45 years.

Dr. C. was well known to the profession by his valuable contributions to the journals—and as the author of several medical works. For a year past he was in charge of the Government Hospitals at Memphis. He was industrious and energetic. By his death the army has lost an efficient officer—the profession an industrious and useful member.

John C. Dalton, M. D., of Lowell, Mass. The medical profession of Massachusetts, and to a certain extent, that of the whole country, have met with a great loss in the death of John C. Dalton, M. D., of Lowell, who died on Saturday, January 9th, 1864, in the sixty-ninth year of his age. Very few medical men who have not been connected with medical teaching or authorship have made themselves more generally known, and still fewer have been as highly esteemed as Dr. Dalton. He was, indeed, the type of *the medical gentleman*, honorable in character, courteous in manners, of integrity that no man doubted or could doubt; amiable and affectionate in all the relations of life. Such men the medical profession can ill spare.

"Peace to a good man's memory."

—*American Medical Times.*

